# Retrospective Exposure Assessment Methods Used in Occupational Human Health Risk Assessment: A Systematic Review

**DOI:** 10.3390/ijerph17176190

**Published:** 2020-08-26

**Authors:** Francesca Borghi, Libero Andrea Mazzucchelli, Davide Campagnolo, Sabrina Rovelli, Giacomo Fanti, Marta Keller, Andrea Cattaneo, Andrea Spinazzè, Domenico Maria Cavallo

**Affiliations:** Department of Science and High Technology, University of Insubria, 22100 Como, Italy; lamazzucchelli@studenti.uninsubria.it (L.A.M.); davide.campagnolo@uninsubria.it (D.C.); sabrina.rovelli@uninsubria.it (S.R.); g.fanti@studenti.uninsubria.it (G.F.); mkeller@studenti.uninsubria.it (M.K.); andrea.cattaneo@uninsubria.it (A.C.); domenico.cavallo@uninsubria.it (D.M.C.)

**Keywords:** exposure assessment, REA, retrospective exposure assessment, occupational exposure, past exposure, JEM, job-exposure matrix, asbestos

## Abstract

As part of the assessment and management of chemical risk and occupational hygiene, retrospective exposure assessment (REA) to chemical agents can be defined as the estimate of exposure associated with a person’s work history. The fundamental problem underlying the reconstruction of the exposure is that of transforming this type of information in quantitative terms to obtain an accurate estimate. REA can follow various approaches, some of which are technically complicated and both time and resource consuming. The aim of this systematic review is to present the techniques mainly used for occupational REA. In order to carry out this evaluation, a systematic review of the scientific literature was conducted. Forty-four studies were identified (published from 2010 to date) and analyzed. In exposure reconstruction studies, quantitative approaches should be preferable, especially when estimates will be used in the context of health impact assessment or epidemiology, although it is important to stress how, ideally, the experimental data available for the considered scenario should be used whenever possible as the main starting information base for further processing. To date, there is no single approach capable of providing an accurate estimate of exposure for each reasonably foreseeable condition and situation and the best approach generally depends on the level of information available for the specific case. The use of a combination of different reconstruction techniques can, therefore, represent a powerful tool for weighting and integrating data obtained through qualitative and quantitative approaches, in order to obtain the best possible estimate.

## 1. Introduction

### 1.1. Background

The process by which chemicals interact with the biological system is characterized by two phases: contact with the biological system (exposure) and actual intake into the biological system (dose). Often, in order to understand the relationship between the exposure to a risk agent and the likelihood of disease, it is necessary to define the exposure to the agent of interest in the exposed subjects. Exposure reconstruction was defined as a quantitative estimate of the amount of substance that reaches the subjects of interest for a given exposure event. Ideally, the results of an exposure reconstruction process are complemented by morbidity and mortality data, for deriving an exposure–response (and/or dose–response) relationship and, therefore, an appropriate risk characterization for the risk agent considered [1]. For a complete and fully representative health impact assessment the human exposure to air pollutants should be ideally evaluated overall, following the exposome concept. The exposome concept concerns the totality of exposure from a variety of both internal and external sources (i.e., chemical and biological agents) or more general “exposure and determinants from conception onward, over a complete lifetime” [2,3,4].

The development of quantitative exposure estimates can follow different methodological approaches, depending on the needs of the evaluation to be carried out. First, if the interest is directed towards personal exposure, the level of exposure must be measured or estimated at the point of contact with the subject (e.g., dermal, inhalation exposure), and the amount of absorbed substance must be estimated. Secondly, if the exposure is associated with a specific scenario, the dose can be estimated or measured and subsequently combined with information relating to the duration of the exposure, in order to refine the estimate. In addition, the dose estimate can be carried out with a retrospective (reconstruction of past exposures) or prospective (estimate of present or future exposures) approach. The prospective approach is typically used for regulatory purposes. The retrospective approach can be used, for example, to characterize exposure of occupational populations exposed for professional reasons to chemicals that are now in disuse. The same approach can also be used to estimate past occupational exposures for an individual (or for a population) in relation to specific tasks, professional roles covered or for the entire working life [1].

Epidemiological studies are often used to validate or confirm the results of the health risk assessment: in the study of occupational diseases, one of the criteria for defining the causal link of the pathology under study is the demonstration of the existence of the relationship with the exposure (or dose) to the causal agent. Chronic occupational diseases (such as some respiratory diseases or cancer) require a long period of exposure and development of the disease due to their onset. The direct measurement of the exposure of each subject for the entire duration of the period of interest would be the most accurate method for assessing exposure, but this approach is essentially impossible to apply in practice. Furthermore, considering the long exposure or latency times that sometimes characterize the onset of occupational diseases, it is objectively unlikely that exposure data will be available for the entire work period. Consequently, epidemiological studies on occupational diseases require an estimate of past exposure through a process called “retrospective exposure assessment” (REA) [1].

The production of exposure estimates, to be used for the studies of the exposure–response relationship, can follow different methodologies: some of these are technically complicated and/or time- and/or cost-consuming. The exposure estimates are generally related to the job performed and perfected based on the whole work history of the subject. In any case, it is necessary to consider a certain number of elements that must be used to obtain a quantitative and detailed retrospective evaluation. The REA approaches may vary from the use of methods based on a simple separation according to the category of work performed (e.g., a dichotomization into “exposed” and “unexposed” workers) to more sophisticated assessments, involving the use of statistical models to provide a quantitative estimate of exposure for each job performed in the work history. The methodology also varies according to the type of epidemiological study. Regardless of the strategy used, the common goal of retrospective exposure studies is to define an exposure estimate that is as accurate as possible, within the limits of the resources available.

### 1.2. Problem Statement

REA is a key component of the risk assessment. In this procedure the degree of interaction between the subject and the hazard that characterize the specific job or even task has to be carefully evaluated. The ability to obtain a reliable assessment of past exposure to a specific hazard is crucial, especially for health outcome and disease associated with the exposure to specific hazards and characterized by a long latency (e.g., the occupational exposure to asbestos fibers and mesothelioma).

The estimates of previous exposure are based on two main key exposure factors (duration of exposure and concentration of the chemical agent). Refinement based on the work history of the examined subject (e.g., jobs performed during the work history), needs to consider, therefore, a certain number of specific elements (work period, type of job, job position, department/workplace, frequency of exposure, change in exposure factors over time, etc.) and exposure determinants (use of personal and collective protection devices, etc.), which should be used to obtain a quantitative and detailed retrospective assessment whenever possible. The evaluation of a scenario no longer existing is a complex task, representing one of the most problematic elements in the risk assessment procedure, and different elements can be a source of additional bias. The data available for the assessment are a crucial factor (i.e., often they can be incomplete, too few or gathered for another purpose). Further, the complexity of the work history of the subject (or the population that is being examined), either for the multitasking or for the variety of the existing hazards, is a problematic aspect to assess [1]. Job exposure matrices (JEMs) and individual expert exposure assessment (IEEAs) can be regarded as classical REA methods. Another way to reconstruct a specific exposure scenario or to gather detailed information to evaluate within job variations, is using a job-specific-questionnaires (known as job specific modules (JMSs)). Every method has its strengths and weaknesses, so experts need to take into consideration the biases in the selected method. Some studies have already shown the potential impact of exposure misclassification on the human health risk assessment process, thus emphasizing the importance of conducting high-quality exposure and dose reconstructions [5,6]. Further, Sahmel et al. [1] outlined that the lack of standardized approaches in exposure reconstruction could be attributable to several reasons. First, validating the results of an exposure reconstruction can be difficult due to the complexity in identifying relevant data for the reliability assessment (i.e., to define the accuracy and precision of the estimates) with the additional risk of introducing uncertainty into the reconstruction results. Then, compounding the issue of exposure reconstruction uncertainty is the lack of consensus regarding the best reconstruction methods to use and the applicability of specific methods to particular scenarios. This lack of standardization stems in part from the unique peculiarities of each scenario of interest, including what information is available as a basis for conducting the reconstruction. As a result, many different types of methods for exposure reconstruction have been used and, quite often, the peer review process has failed to ensure that sufficiently robust exposure reconstructions have been routinely conducted [7,8]. Standardized exposure reconstruction methods that incorporate both quantitative and qualitative uncertainty analyses and validation techniques have not been adopted by any regulatory agency or scientific body. Recent studies provided helpful information, but did not present a detailed review of exposure reconstruction methods as they have been used in the peer-reviewed literature [9,10], while the latest systematic review on the topic was published in 2010 by Sahmel et al. [1].

### 1.3. Aim of the Study

The aim of this systematic review is to present the techniques mainly used for occupational REA. In more detail, this study aims to (i) identify which scenarios have been considered in REA studies (both in terms of jobs/activities and chemicals used); (ii) report methods (with particular emphasis on the statistical methods) used for REA; (iii) report the main parameters to be considered in these evaluations; (iv) list the principal JEMs used in the methodological studies under investigation; and (v) list the problem and solution related to the past exposure reconstruction found by the authors. Since REA is such a crucial factor for workers exposed to asbestos fibers, this review is also aimed at addressing the main problematics of this field evaluating which aspects are more relevant for a reliable REA. Since REA is such a crucial factor for workers exposed to asbestos fibers, which is an emblematic case of application of the REA (since occupational exposure to asbestos was widespread in many sectors and for different categories of workers, and can lead to severe pathologies after a long latent period) this review is also aimed at addressing the main problematics of REA applied to occupational exposure to asbestos fibers in particular.

## 2. Materials and Methods

This study involved a systematic review process conducted according to the Preferred Reporting Items for Systematic Reviews and Meta-Analyses Statement criteria (PRISMA) [11]. Both retrospective occupational exposure assessments in general and REA for asbestos workers (REA–Asbestos) have been considered. The systematic review was conducted using the outcomes from three different databases (i.e., Scopus, Web of Science and PubMed). The databases were analyzed using the same queries of search, that were arranged in order to match the syntax rules required by each database. Two different queries of search were used for (i) the REA in general (Table 1) and for the (ii) retrospective exposures to asbestos (Table 2).

**Figure 1:**
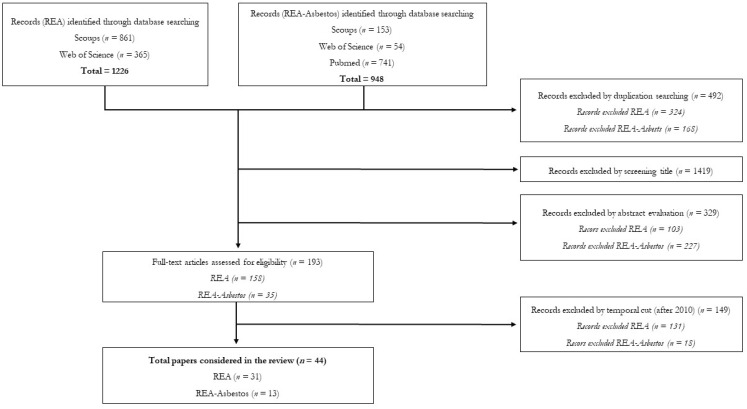
Systematic review flowchart, modified from Moher et al., 2009 [11].

As reported in Figure 1, a total of 1226 articles were retrieved for REA in general, of which 861were from Scopus and 365 from Web of Science. For the REA concerning occupational asbestos exposure, a total of 948 articles were found (of which 153 in Scopus, 54 in Web of Science and 741 in Pubmed). After the elimination of the duplicates and the evaluation of the coherence of the title with the aims of the review, a total of 902 articles for the REA and 780 for the REA–Asbestos were included in the review. The evaluation of the titles’ coherence was conducted by three experts, in order to reduce the error associated to the operator and to operate as rigorously as possible. A total of 103 papers for the REA and 227 for REA–Asbestos were subsequently excluded based on their abstract and the affinity with the review. In more detail, the inclusion criteria included original, peer-reviewed articles, published in English, and reporting methods and procedures for REA and REA–Asbestos. Exclusion criteria included case reports, conference papers, and publications that did not focus on occupational exposure (for this reason, both studies regarding environmental or consumer exposure were not considered in this review), or that were published in languages other than English. Finally, all scientific papers written before 2010 have been discarded, as they were already considered by [1]. As a result, 104 papers for the REA and 18 for the REA–Asbestos were excluded and a total of 44 papers were reported in the present review; the last research was conducted the 19 May 2020 (weekly updates of the research in scientific databases were performed until the date of submission of the manuscript, anyway).

## 3. Results and Discussions

As reported in the “Materials and Method” section, a total of 44 articles concerning methods used in the REA were found. The complete list is provided in Table 3.

The results of the eligible studies are described in the following sections derived for REA (in general) and for REA–Asbestos studies. The following paragraphs present and discuss an examination of the following aspects, as reported in the eligible studies: (i) the considered occupational exposure scenarios; (ii) the REA methods used; (iii) the parameters considered for the REA; (iv) the statistical methods; (v) the JEMs used; as well as (vi) the results obtained; and (vii) the problems identified and the solutions proposed.

### 3.1. REA

#### 3.1.1. Applications for Occupational Exposure to Chemical Risk Agents

Exposure scenarios interested by REA and considered in this review are numerous and heterogeneous. Workers in the automobile industry [39], in the sand [47] and in rubber industries [31,32], as well as mining workers [53,55] and military veterans [15] were considered in this work. Moreover, results from chemical industry studies were reported [18,21,25,33,35,41,49,54]. As for the activities involved, a wide variety of chemical hazards were considered in the REA assessment and are summarized in Table 4. REA applications for occupational exposures to asbestos are reported in Section 3.2.

#### 3.1.2. Methods Used in the Past Exposure Reconstruction

As stated before, REA is a complex task, the execution of which can be done following different methods and techniques. Every method used for REA is characterized by its own pros and cons, so to obtain the best possible representation of a specific scenario, these methods can be mutually combined to improve the reliability of estimates. Moreover, the availability of well-structured and complete databases is a critical aspect in the REA process, and, when these data are available, both qualitative and quantitative methods can be used. For example, the reconstruction of exposures in epidemiological analysis would be facilitated in cases where specific databases containing historical series of monitoring data and contextual information are pre-established [53]. Nevertheless, data availability may not comply with the purpose or with the standards of the REA process. For this reason, simulation algorithms can be used to identify a proper conversion factor to adapt data to the standards of the study [47]. A common practice, that involves the use of measurements and contextual data of a specific scenario or task, is the grouping of workers with a similar exposure profile into similar exposure groups (SEGs), allowing one to obtain a picture of the presence or absence of the risk agent in the SEGs and an exposure estimation [49]. As reported in the literature [41], the average levels of exposure of a specific group can be applied to all workers included in that group. For the REA of a specific scenario, industrial hygienists usually use information about the characteristics of the working scenario (i.e., industry, job title, task performed, materials involved, etc.) to express in a qualitative (yes/no) or semi-quantitative (exposure categories) way their expert judgement about the level of exposure [15]. The use of statistical models to assign estimated exposure intensities is a reliable practice that allows the development of quantitative and more reproducible methods for REA [34]. For example, hierarchical models can be used in order to obtain clusters of data concerning a specific factory, task or job [43,44,45]. Finally, questionnaires are a useful tool to gather a-posteriori information about a certain exposure scenario [13], especially when these can provide information about every task held by the subject in his working schedule.

#### 3.1.3. Parameters to Be Considered

In order to correctly assess the retrospective exposure of a specific cohort, different parameters need to be considered for every job held by the subject whose work history is being analyzed. In addition to some more general parameters, which are already discussed extensively in Sahmel et al. [1], some specific parameters to consider are shown below. Aside from those listed below, other parameters may be necessary, depending on the type of study being conducted and the exposure scenario considered [1].In particular, the following issues should be carefully addressed in REA studies: type of industry, job title, length of the employment, task performed and materials used for each tasks [15], information from plant personnel and documentations obtained from the industry historical databases [21], the use of collective protective measures and personal protective equipment’s (PPE) [41], possible sources of non-occupational exposure [13], material safety data sheets, summary of process changes and process specification, lab-tests for possible process intermediate, accident and injury report, changes of the department during the employment, movement of the employee among the process and the plant [31].

The temporal aspect of the exposure is also crucial in the REA process: duration, frequency and distribution of the exposure related tasks (daily, weekly and monthly) [27] should be taken into account. Moreover, boundary information, such as weather characteristics, proximity to a major road and land use, can be considered while assessing the exposure of large cohorts [24]. Finally, characteristics of the working place (i.e., local exhaust ventilation, industrial mechanical dilution, location of the working area, proximity to the source, process temperature and confinement of the space [34]) can be also relevant for the study, as well as information regarding health status of the subject, such as the smoking status [24].

#### 3.1.4. Statistical Methods

Common statistical methods used in the REA assessment were different: for example, the mean concentrations were used to represent the exposure of a specific SEG or a specific job [53] while the arithmetic mean of the data over time (where the exposure was judged to be stable) and mean values of exposure across the time period when there were no statistical changes in the exposure were used by Rando and collaborators [47]. Mean exposure intensity [49], average concentrations [41] and central values of the exposure probability of different categories [27] were also used. Daily Weighted Average (DWA), calculated for each job, and both arithmetic and geometric mean exposure were evaluated in order to provide data for a sensitivity analysis by Couch [21]. Moreover, the odds ratios (ODs) were used by Mester and collaborators [39] to evaluate the level of disagreement between different ratings of exposure. Other statistical techniques, such as linear regression models [55], least-square linear regression analysis for the evaluation of data collected from personal and area samplings [54], Bayesian hierarchical models [50] and weighted Cohen’s Kappa with linear weights [36] as well as Structural Equation Modelling Techniques (SEM) for predicting shifts in the exposure [24] were used. For the evaluation of models, methods such as Cross-validation of the modelling process were applied using data splitting and Monte Carlo techniques [34].

#### 3.1.5. JEMs Used

Different JEMs were used for the REA of different work scenarios. For example, Mester and collaborators [39] used an industry-specific JEM while Rando [47] developed a JEM combining data from a modern Respirable-Quartz database and archival particulate count exposure data. Couch [21] designed a JEM for the exposure assessment to beryllium, like the one designed by Sivulka and collaborators [54] for the evaluation of the exposure to nickel in a cohort study and the one from Canu et al. [33] to evaluate Uranium exposure. Moreover, JEMs specific to one agent has been developed by Févotte [27] to estimate of the probability and level of exposure in a general population study. A job exposure matrix for specific agents (both for chemical and physical agents), such as asphalt fume, total particulate matter, respirable crystalline silica, benzene, formaldehyde, 1,3 butadiene, as well as ionizing radiations, was also developed [25,35].

#### 3.1.6. Principal Results

Comparing different methods for the REA, Mester and collaborators [39] reported that the most important determinants for disagreement between a JEM and an expert panel for the exposure assessment (EA-panel) were: (i) inability to assess tasks conducted outside the production line (disagreement 80%); (ii) low probability of exposure (disagreement 25%) and (iii) exposure depending on specific task, in the production line, that required the usage of specific chemicals, lacquers in this case (disagreement 32%). Using different approaches for the evaluation of the same scenario can lead to different results: there is the need to take these differences into consideration during the REA, wherever possible, and approaches based on databases with quantitative and detailed information, as well as time-trends, should be preferred [53].

The expert’s judgment is considered, as seen previously, the golden standard technique for the assessment of past scenarios, but, as suggested by Bello et al. [15], when there is a lack of detail about a specific industry or job, the “raters” (i.e., industrial hygiene experts, each individually with >20 years of experience conducting workplace hazard assessments) tend to be conservative causing an overestimation of the risk of exposure. The same job task, evaluated in different time periods, can show differences in the exposure levels due to application of personal protection devices, such as respiratory protections, suggesting that time reference of data are key factors that need to be considered when assessing the exposure in a retrospective way [47]. Decrease of the exposure levels was also observed by Rodrigues et al. [49], stating that mean exposure, in that case between semiconductor and storage device manufacturing workers, has decreased by an order of magnitude over the three manufacturing periods considered in the study. Other authors observed a reduction of the exposure in different scenarios [25,31,32,54,55].

The identification of jobs potentially exposed to a certain hazard is a crucial factor in REA that allows the designation of SEGs and the identification of critical tasks; oftentimes, the examined scenarios are numerous and techniques, such as crosswalk, for the translation of job codes, are usually applied. In particular, Koeman and collaborators [36] reported in their study that crosswalk translations are comparable to the ones conducted with a manual recording.

Some specific characteristics of the exposure scenario can affect the levels of risk of exposure for certain subjects: Fleming [28] reported that workers with the same job title had different potential exposure due to minor changes in the working-schedule. Further, in their study, Canu and collaborators [33] observed that workers who handled uranium compounds with low solubility had a higher relative risk of lung cancer.

#### 3.1.7. Problems and Solutions

The amount and quality of data available for the development of JEM is usually a crucial element in REA studies: data may be too few for a categorization of workers by exposure levels [33], the time period over which the data were collected may be unsuitable for the purpose of the study [45] and the data may not be enough for developing a quantitative cumulative exposure matrix or not detailed enough for a good characterization of a specific scenario [28]. The REA conducted using data from a specific occupation or job title is a source of well-established and detailed information of an exposure scenario. However, sometimes there is the risk of misclassification of risk agents and the multiple sources of variability within a job are a crucial factor, especially when assessing cumulative exposure. Minor errors in the estimation can result in a substantial misclassification affecting the ability to assess correctly the degree of exposure for a specific case-study. Heterogeneity of working stations [27] and temporal changes in the exposure to a certain hazard and in the exposure factors are other sources of bias, particularly in chronic epidemiological studies [24]. Moreover, the effectiveness of respirators at workplace may vary significantly from the nominal protection factor, from day-to-day, and across workers [21].

For the reasons reported above, future efforts should be made to incorporate exposure variability estimates into uncertainty analyses, as suggested by the literature [23].

Obviously, unavailable or incomplete data are a common problem for the characterization of job scenarios in REA: to solve this problem usually assumptions and predictions (based on similar scenarios) are used but this may introduce, somewhat, an uncertainty in the exposure estimates. The problem of the missing data is described by Sauvé and collaborators [51], who stated that missing data were prevalent in some studies for several variables (i.e., use of control methods, protective equipment) during the exposure assessment phase, based on job descriptions. Moreover, the missing data concerning comprehensive external data set may lead to the missing validation of the exposure models: this is a common limitation of most models developed for REA, as reported in the literature [30].

Using questionnaires specific for a determined job task is a useful tool to obtain detailed information that is not otherwise available. These questionnaires must be designed in a specific way in order not to introduce errors due to the misunderstanding of the employees. Therefore, sources need to be reinvented using terms familiar to the workers. Another source of error while assessing the exposure in a retrospective manner is the misidentification of hazards: for example, some Endocrine Disruptors Chemicals (EDC), which are present in a great variety of substances at work, are rarely and unlikely being recalled by the majority of the employees due to the complexity of the job schedule [39].

IEEA is a common practice for the estimation of past exposure that relies on the knowledge and the experience of the assessor when there are no actual exposure measurements. A big concern about IEEA methods is the reliance only on expert knowledge and experience about a specific scenario of exposure: the variety of industries and occupations characterizing population-based studies is a source of uncertainty for the assessor and can result in a misclassification and misidentification of the exposure intensity [34]. Despite being the reference method for the REA in community-based studies, thanks to the variety of data and information that are taken into consideration for assessing a specific scenario, the IEEA is a taxing and resource-demanding process, especially when applied to large population studies, so its applicability is limited.

Finally, automatic translations of job-coding are a useful technique whenever there is the need to adapt a large database to a different classification-system; the drawback of this technique is that it is strongly influenced by subjectivity (since most cross-walk translations are conducted by a single expert) and the absence of a gold standard for the job codes or exposure estimates to be referred to [36]. As reported in another study, indeed, workers may be not correctly classified into the correct categories and, depending on the magnitude and direction of the exposure misclassification, this could have significant implications on the epidemiological findings [18].

From the results reported before, it is evident how the retrospective assessment of exposures requires many assumptions that lead to uncertainty in the exposure estimates across workers [24].

### 3.2. REA–Asbestos

#### 3.2.1. Applications for Occupational Exposure Assessment to Asbestos

Since asbestos have been widely used in different industrial scenarios, the cluster of activities interested by an REA of asbestos fibers is broad and various. In this review different industrial scenarios were considered, such as the power industry [26], iron foundries [12] and others [16,19,20,29]. As is well known, the target organs for asbestos are the lungs and the lung mucous membranes. Therefore, besides asbestos fibers (also considering the polymorphisms), agents with the same target organ such as chromium, nickel, Polycyclic Aromatic Hydrocarbons (PAH), Respirable Crystalline Silica (RCS), silicon carbide, quartz, cement, coal and respirable dust/fibers were evaluated [29,42,43,46].

#### 3.2.2. Methods Used in the Past Exposure Reconstruction

Developing a quantitative exposure matrix is a common practice for the evaluation of exposure to asbestos; these matrices can be (i) a general population matrix (GPJEM) [20]; (ii) quantitative job-exposure matrix (JEM) [42] or (iii) developed on statistical modelling [44]. Since long latency is a characteristic of the asbestos’ exposure, a crucial factor is the ability to predict historical and current exposure patterns: for this reason mathematical models were developed [12]. Moreover, specifically designed tools for the assessment of the exposure to asbestos, such as OccIDEAS [38], were developed using both historical and self-reported information’s (throughout questionnaires). It is important to underline that different JEMs can be compared with the expert assessment (considered the golden standard, as far as REA concern) to evaluate their efficiency and their reliability [42].

Furthermore, in the case of REA–Asbestos, data availability is a limitation in the retrospective exposure process, as stated before. Adapting and restoring databases created for a different goal is a practice that allows the evaluation of historical exposure [22] or to gather pertinent exposure measurement data through the identification of job titles considered exposed to asbestos fibers [43]. Data on self-reported exposure, obtained through questionnaires, are used in cohort studies to obtain information on subjects linked by the same exposure scenario [26].

Finally, biological monitoring the exposure to asbestos fibers is, in most cases, a good indicator of the effectiveness of the exposure. Therefore, techniques such as the experts’ judgements, can be tuned and compared with pathology assessment in order to assess the reliability of the technique [48].

#### 3.2.3. Parameters to Be Considered

Since the asbestos fibers are an airborne agent, different parameters (i.e., sampling period and duration, collected air volume, concentrations in air, site descriptions and locations) needs to be evaluated [22]. Besides job titles, length of the exposure and specific occupational tasks [26] and a priori exposure rating for each held job (non/low/high exposed) [44] should be preferably included in the assessment, in order to analyze the scenario with greater detail. Moreover, data regarding the substance emission potential, the activity emission potential and effectiveness of any local control, as well as the passive and/or fugitive emissions and the efficiency of any respiratory protection (RPE) [19] are relevant in the assessment procedure. Finally, personal conditions (i.e., depression, anxiety), can also affect the ability of a subject to evaluate his working conditions while undertaking a job-specific-questionnaire or habits (i.e., smoking habits) and, for this reason, these personal conditions can interfere with the assessment procedure [46].

#### 3.2.4. Statistical Methods

Exposure levels may be expressed by Weighted Arithmetic Mean (WAM); the Arithmetic Mean (AM) was used as a representative value for the analysis as it is considered the best summary value for chronic diseases [20]. The exposure levels may be expressed as the product of the eight-hour time weighted average and as the total duration of exposure in in fiber concentration per years (fibers/cm^3^/years) [26], as well as by Time-Weighted averages (TWAs). To evaluate the correlation between different techniques, both regression and ANCOVA analyses [48] were used.

#### 3.2.5. JEMs Used

JEMs specifically used in REA to asbestos were: (i) GPJEM, developed on Korean domestic quantitative exposure datasets [20]; (ii) SYN-JEM, a JEM developed to assess the exposure to five major lung carcinogens [43] which was then extended into (iii) a quantitative JEM that uses personal occupational exposure measurements [44]. Offermans et al. [42] performed a comparison between three different JEMs (DOMJEM (asbestos, PAHs), FINJEM (asbestos, PAHs and welding fumes) and Asbestos JEM (asbestos)) to evaluate their reliability.

#### 3.2.6. Principal Results

The scenarios considered at risk of occupational exposure to asbestos fibers changed throughout the years; for example, as reported by Choi and collaborators [20] who assessed the exposure to asbestos fibers with a General Population JEM, in the 1980s the highest exposure levels were estimated in the “knitting and weaving machine operators”, in the 1990s the most exposed sector was the “plastic products production machine operators” and in the 2000s the “detergents production machine operator” was the most exposed. Moreover, specific materials, such as lay-in ceiling panels containing asbestos fibers, can have different intensity magnitudes, depending on the category to which the subject who is handling the materials belongs; indeed, specialists were found to have the greatest exposure, followed by maintenance workers, generalists, bystanders and DIY [17].

The ability to assess the retrospective exposure and its intensity is a crucial factor, especially for lung carcinogens characterized by a long time of latency. SYNJEM [44], result to be a valid instrument for the assessment of occupational exposures in a quantitatively-way. Regarding the evaluation of the models, Cherrie and collaborators [19] evaluated in their study the efficiency of a model to estimate past exposure to asbestos, finding out that despite an overestimation at lower levels and an under-estimation at higher levels, results obtained with the model were comparable with measured exposure levels.

#### 3.2.7. Problems and Solutions

JEMs developed on large databases, such as GPJEM, based on domestic quantitative exposure data covering the major industries in the Republic of Korea, can be used as a surveillance system [20]. Large databases, similar to the ones collected by governments, can be a good source of measurements underlying historical trends, such as the (i) application of regulatory limits; (ii) improvements in the appliance of improved working-practice or (iii) the introduction of control methods; other than the (iv) improvements in the sampling techniques. A subject’s working history is such a unique scenario (and one which often shows significant changes over time in terms of exposure conditions) that can rarely be characterized, with a good level of detail, with REAs. Chung and collaborators [22] found that the major source of variability, relating to the use of large databases for a retrospective assessment, is the large range in the exposure measurements because random and systematic errors are a huge source of variability. Moreover, available data could be (i) collected for a different task, with a non-standardized technique or (ii) the information’s could be reported in different language and/or coding systems, creating more bias that may interfere with the accuracy of the assessment [43]. Estimations developed on specific materials or chemicals tend to not consider other sources of probable asbestos exposure [16]: in this way the exposure level in complex working-scenarios, is usually under-estimated. Moreover, the necessity of the assessors’ training, as well the use of two or three independent assessors in exposure reconstruction, is useful in the reduction of the variability in the final estimates [19]. Further, since lung cancer is a pathology characterized by a long latency, the exposure assessment is a complex procedure because the exposure may have happened years before the assessment. Further complications may come from a lack of information or detailed measurements regarding the evaluated scenario, assumption and estimations need to be taken in order to evaluate the nature of the exposure, but they can be also a source of bias. Finally, there are some limitations of the time trend approach, specifically where there is large confidence interval around the time trend values: in this case the major source of this variability refers to the large range in the exposure measurements [22]. As stated in another study [42], lack of a time axis could also be responsible for the variable agreement, because exposure levels have generally decreased over time because of better understanding of occupational hazards and subsequent regulations.

## 4. Conclusions

REA is a crucial component of the risk assessment procedure when the evaluation of the past occupational exposure to chemicals and other hazards is needed in large population-based studies or in specific working-scenarios. The ability to evaluate the level of past exposures for a cohort of workers is a key element for the early detection of pathologies characterized by a long latency time, e.g., for the occupational exposure to asbestos fibers. Each assessment method that can be used to reconstruct the exposure of a past scenario has his own advantages and disadvantages that need to be taken into account when assessing the exposure and analyzing the outcomes. REA procedures can be applied to a wide variety of exposure-scenarios, either for population-based studies, evaluating the level of exposure to a certain hazard, or cohort studies linked by the same task or the professional use of a specific risk agent. Multiple hazards, especially those characterized by a large field of usage, and consequently involving many workers, or those with a peculiar toxicokinetic profile causing a late onset of the pathology (which is a common feature of lung carcinogens) can be effectively analyzed with an REA procedure.

In order to correctly assess the level of exposure in past exposure scenarios, there is the need for well-structured exposure databases. The analysis and description of a scenario that is no longer present is often a challenging task that needs, as an input, a detailed description of the performed job or tasks and much other contextual information. However, in most cases, the lack of data obliges one to assess the exposure by predictions and assumptions that can led to errors in defining a reliable exposure estimate and, thus, may lead to incorrect conclusions on the identification of subjects actually exposed to a certain agent.

In a complex working-scenario, elements, such as the multiple tasks carried out within a job, multiple materials and chemicals handled during the working process (also considering possible secondary products that can be released during the production), personal factors and personal habits (which can be a source of exposure during extra-occupational activities), are key elements for a reliable reconstruction of past exposure scenarios. For the purpose of obtaining a more detailed and reliable REA, there is the need to develop systematic and defined procedures for the gathering of information and the creation of specific databases based on well-characterized exposure scenarios for many risk agents.

## Figures and Tables

**Table 1:** Query used for the retrospective exposure assessment (REA) in general. N: number of papers results from the search. Date of search: 18 May 2020.

General Retrospective Assessment		
Database	Search Query	*n*
Scopus	(TITLE-ABS-KEY(“exposure assessment” OR “occupational exposure” OR “risk assessment” OR “exposure estimation”) AND TITLE-ABS-KEY(“exposure reconstruction” OR “past exposure” OR “historical exposure” OR “retrospective exposure assessment”))	857
Web of Science	TS = (“exposure assessment” OR “occupational exposure” OR “risk assessment” OR “exposure estimation”) AND TS = (“exposure reconstruction” OR “past exposure” OR “historical exposure” OR “retrospective exposure assessment”)	361
Pubmed *	(((((exposure assessment) OR occupational exposure) OR risk assessment) OR exposure estimation)) AND ((((exposure reconstruction) OR past exposure) OR historical exposure) OR retrospective exposure assessment)	11,800

* Due to the high number of articles obtained using this search query in Pubmed, it was decided not to consider this database for this part of the review.

**Table 2:** Query used for REA studies dealing with asbestos. N: number of papers results from the search. Date of search: 18 May 2020.

Retrospective Assessment of Asbestos		
Database	Search Query	*n*
Scopus	(TITLE-ABS-KEY(“exposure assessment” OR “occupational exposure” OR “risk assessment” OR “exposure estimation”) AND TITLE-ABS-KEY(“exposure reconstruction” OR “past exposure” OR “historical exposure” OR “retrospective exposure assessment”)) AND TITLE-ABS-KEY (asbestos)	152
Web of Science	TS = (“exposure assessment” OR “occupational exposure” OR “risk assessment” OR “exposure estimation”) AND TS = (“exposure reconstruction” OR “past exposure” OR “historical exposure” OR “retrospective exposure assessment”) AND TS = (asbestos)	51
Pubmed	(((((exposure assessment) OR occupational exposure) OR risk assessment) OR exposure estimation)) AND ((((exposure reconstruction) OR past exposure) OR historical exposure) OR retrospective exposure assessment) AND (asbestos)	730

**Table 3:** Articles considered in this review.

Reference	Author	Year of Publication	Contents
[12]	Andersson et al.	2012	**
[13]	Armstrong et al.	2011	*
[14]	Banerjee et al.	2014	*
[15]	Bello et al.	2017	*
[16]	Boelter et al.	2016	**
[17]	Boelter et al.	2017	**
[18]	Chen et al.	2012	*
[19]	Cherrie et al.	2018	**
[20]	Choi et al.	2017	**
[21]	Couch et al.	2011	*
[22]	Chung et al.	2015	**
[23]	Dopart et al.	2017	*
[24]	Davis et al.	2011	*
[25]	Fayerweather et al.	2011	*
[26]	Felten et al.	2010	**
[27]	Févotte et al.	2011	*
[28]	Fleming et al.	2014	*
[29]	Føreland et al.	2012	**
[30]	Friesen et al.	2012	*
[31]	Hanley et al.	2012	*
[32]	Hidajat et al.	2019	*
[33]	Canu et al.	2010	*
[34]	Johnson et al.	2017	*
[35]	Jones et al.	2015	*
[36]	Koeman et al.	2013	*
[37]	Lavoué et al.	2020	**
[38]	MacFarlane et al.	2012	**
[39]	Mester et al.	2011	*
[40]	Miller and Machado	2010	*
[41]	Nano et al.	2012	*
[42]	Offermans et al.	2012	**
[43]	Peters et al.	2012	**
[44]	Peters et al.	2016	**
[45]	Portengen et al.	2016	*
[46]	Lin et al.	2014	**
[47]	Rando et al.	2018	*
[48]	Rasmuson et al.	2014	**
[49]	Rodrigues et al.	2019	*
[50]	Sauvé et al.	2019	*
[51]	Sauvé et al.	2019	*
[52]	Sauvé et al.	2018	*
[53]	Shao et al.	2019	*
[54]	Sivulka et al.	2014	*
[55]	Vermuele et al.	2010	*

* Articles concerning the REA in general; ** Articles concerning the REA–Asbestos.

**Table 4:** Summary of chemicals considered in the studies under review.

Reference	Chemicals
[53]	Elongate mineral particle (EMP)
[25,47]	Respirable quartz
[55]	Respirable Elemental Carbon (REC) and carbon monoxide (CO)
[15]	“Group A” agents (lead, formaldehyde, hydrocarbon solvents, and chlorinated solvents) and “Group B” agents (mercury, selenium, arsenic, polychlorinated biphenyls, electromagnetic field, pesticides, and viral agents)
[39]	Endocrine Disruptors Chemicals (EDC)
[41]	Polycyclic Aromatic Hydrocarbons (PAH)
[13,30,45]	Benzene
[21]	Beryllium
[54]	Nickel
[50]	Wood dust
[25]	Asphalt fumes and total particulate matter
[32]	Rubber dust, rubber fumes and n-Nitrosamines
[18,36,37,45,52]	Other chemicals
